# Regulation of Toll-like receptor 4-mediated immune responses through *Pasteurella multocida* toxin-induced G protein signalling

**DOI:** 10.1186/1478-811X-10-22

**Published:** 2012-08-01

**Authors:** Dagmar Hildebrand, Aline Sahr, Sabine J Wölfle, Klaus Heeg, Katharina F Kubatzky

**Affiliations:** 1 Department für Infektiologie, Medizinische Mikrobiologie und Hygiene, Im Neuenheimer, Feld 324, D-69120, Heidelberg, Germany

**Keywords:** Monocytes, Toll-like receptor 4, Heterotrimeric G proteins, *Pasteurella multocida* toxin, Interleukin-12, T lymphocytes, Immune evasion

## Abstract

**Background:**

Lipopolysaccharide (LPS)-triggered Toll-like receptor (TLR) 4-signalling belongs to the key innate defence mechanisms upon infection with Gram-negative bacteria and triggers the subsequent activation of adaptive immunity. There is an active crosstalk between TLR4-mediated and other signalling cascades to secure an effective immune response, but also to prevent excessive inflammation. Many pathogens induce signalling cascades via secreted factors that interfere with TLR signalling to modify and presumably escape the host response. In this context heterotrimeric G proteins and their coupled receptors have been recognized as major cellular targets. Toxigenic strains of Gram-negative *Pasteurella multocida* produce a toxin (PMT) that constitutively activates the heterotrimeric G proteins Gα_q_, Gα_13_ and Gα_i_ independently of G protein-coupled receptors through deamidation. PMT is known to induce signalling events involved in cell proliferation, cell survival and cytoskeleton rearrangement.

**Results:**

Here we show that the activation of heterotrimeric G proteins through PMT suppresses LPS-stimulated IL-12p40 production and eventually impairs the T cell-activating ability of LPS-treated monocytes. This inhibition of TLR4-induced IL-12p40 expression is mediated by Gα_i_-triggered signalling as well as by Gβγ-dependent activation of PI3kinase and JNK.

Taken together we propose the following model: LPS stimulates TLR4-mediated activation of the NFĸB-pathway and thereby the production of TNF-α, IL-6 and IL-12p40. PMT inhibits the production of IL-12p40 by Gα_i_-mediated inhibition of adenylate cyclase and cAMP accumulation and by Gβγ-mediated activation of PI3kinase and JNK activation.

**Conclusions:**

On the basis of the experiments with PMT this study gives an example of a pathogen-induced interaction between G protein-mediated and TLR4-triggered signalling and illustrates how a bacterial toxin is able to interfere with the host’s immune response.

## Background

Monocytes are professional antigen presenting cells and carry out at least two important functions during infection. First of all they represent a barrier against pathogens through their antimicrobial activity and second they support the initiation of adaptive immune responses. The latter is exerted by the presentation of processed antigens on major histocompatibility complex (MHC) molecules to T lymphocytes, the expression of various costimulatory proteins on the cell surface and the production of cytokines [1,2]. To direct these functions during an immune response, monocytes become activated through binding of conserved microbial structures to their respective pattern-recognition receptors (PRRs). Within the group of PRRs, Toll like receptors (TLRs) play a well-known role in the initiation of such immune responses. Up to now, 10 functional TLRs have been identified in humans [3]. Each TLR detects distinct PAMPs derived from viruses, bacteria, mycobacteria, fungi, and parasites [3]. Gram-negative bacteria are typically sensed through the cell wall constituent lipopolysaccharide (LPS) that binds in complex with the LPS-binding Protein (LBP) to a receptor complex of TLR4, CD14 and an associated protein (MD-2) [4]. The TLR4-mediated signalling cascades then modulate the gene expression towards the production of a variety of pro-inflammatory cytokines such as Interleukin (IL)-6, Tumour necrosis factor (TNF)-α and IL-12 [5]. In addition, these signalling events enhance the costimulatory function of monocytes [6]. However, this TLR4-mediated induction of inflammation and activation of adaptive immunity is actively targeted and modulated by a variety of pathogens, presumably to impair the immune response. At this, heterotrimeric G proteins play an important role. Bacterial toxins that act on heterotrimeric G proteins such as Cholera Toxin (CT) of *Vibrio cholerae*, heat-labile enterotoxin (LT) of enteropathic *Escherichia coli* or Pertussis Toxin (Ptx) of *Bordetella pertussis* can therefore alter LPS-induced cytokine release. This is achieved by heterotrimeric G protein-mediated production of cAMP which eventually results in an altered cytokine release, most notably of IL-12, by human monocytic cells. The molecular mechanism of the effect is however only incompletely understood [7,8].

Another toxin that targets heterotrimeric G proteins is produced by toxigenic strains of Gram-negative *Pasteurella multocida* bacteria that can be isolated from chronic respiratory infections in animals and from humans after cat or dog bites [9]. *Pasteurella multocida* toxin (PMT) is known to activate Gα_q_, Gα_i_ and Gα_13_[10-13] independently of a G protein-coupled receptor through deamidation resulting in the constitutively activation of the α-subunit and release of the βγ-subunit [14]. This leads to the downstream activation of signalling events such as phospholipase Cβ activation, induction of the mitogen-activated protein (MAP) kinase pathways, the RhoA/Rho kinase (ROCK) pathway and the Janus kinase (JAK)-signal transducers of transcription (STAT) pathway [15-18]. Additionally, PMT activates Gα_i_, thus inhibiting adenylate cyclase activity and cAMP accumulation [10].

In this study we investigated how the activation of heterotrimeric G proteins through PMT influences the TLR4-mediated activation of human blood-derived monocytes (hBDMs) and their ability to induce T cell proliferation. Our data demonstrate that PMT modulates TLR4-mediated cytokine production. This effect was most pronounced for the release of IL-12p40, a cytokine important for T cell activation. The suppression of IL-12p40 release resulted in the inhibition of the T cell-activating ability of LPS-activated hBDMs. This block could be restored by adding IL-12-containing supernatants of LPS-stimulated hBDMs to the mixed lymphocyte reaction (MLR), showing that IL-12 was both, necessary and sufficient. The toxin mediates this inhibition of TLR4-induced IL-12p40 expression by Gα_i_-mediated inhibition of adenylate cyclase and subsequent cAMP accumulation in combination with a Gβγ-mediated activation of PI3kinase and the MAP kinase JNK. These findings give new insights into the modulation of an immune response by a bacterial toxin and we hypothesise that the decreased T cell activation results in increased survival of the pathogen.

## Results

### PMT modulates LPS-triggered cytokine release of human blood-derived monocytes

Antigen presenting cells regulate lymphocyte-mediated immune responses through presentation of antigens to lymphocytes via the major histocompatibility complex (MHC) on their surface, costimulatory or co-inhibitory signals that are delivered by ligands of the B7 family and via the release of cytokines [19].

**Figure 1:**
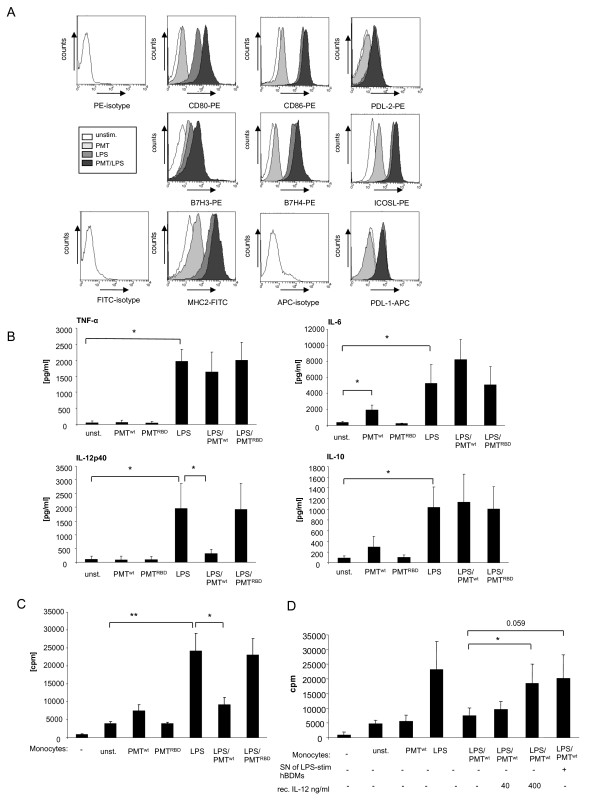
**PMT-mediated modulation of LPS-activated monocytes.** A. Monocytes were stimulated with PMT^wt^ (1 μg/ml, 3 h prestimulation) and/or LPS (30 ng/ml; over night stimulation) or left unstimulated. The expression levels of surface markers were analysed by flow cytometry using fluorescently labelled antibodies against CD80, CD86, B7H3, B7H4, ICOSL, MHC2, PDL-1 and PDL-2. The results are presented as overlays which were produced using Weasel v2.5 software. Shown is one representative result from three independent experiments. B. Cells were stimulated as described in A. As additional control inactive PMT^RBD^ (1 μg/ml, 3 h prestimulation) was used. The release of TNF-α, IL-6, IL-10 and IL-12p40 was measured by ELISA. Shown is the mean of three independent experiments (mean ± SD; n = 3) C. Monocytes were stimulated as described in A, washed with media and used for MLR with 1x10^4^ CD14^+^ hBDMs and 1x10^5^ CD3^+^ alloreactive T cells. After three days of co-culture proliferation of T cells was analysed by [^3^H]-thymidine incorporation and presented as counts per minute (cpm). D. Cells were stimulated and T cell proliferation was analysed as described in C. To rescue the T cell-activating ability of LPS/PMT-treated monocytes, co-cultures were supplemented with supernatant (Sn) of LPS-treated monocytes or recombinant IL-12. MLRs were performed in triplicates. Shown is the mean of three independent experiments (mean ± SD; n = 3). Statistical significance was assessed by using student’s *t* test with *P < 0.05.

Our study aimed to clarify whether the LPS-induced activation of hBDMs through TLR4 is modulated by the heterotrimeric G protein activator PMT and whether the toxin itself activates monocytes. We prestimulated hBDMs with wildtype PMT (PMT^wt^) or the inactive mutant PMT^RBD^, which contains the receptor binding domain (RBD) but lacks the C-terminal catalytic domain (CD), for three hours, as the intracellularly-acting toxin needs time to enter the cell and to translocate to the cytoplasm [20]. LPS was then added in co-stimulated samples for overnight incubation. The first experiments addressed whether the expression of immune-relevant ligands on monocytes was changed in the presence of PMT compared to unstimulated or LPS-treated samples. The surface expression of MHC II and the B7 family members B7.1 (CD80), B7.2 (CD86), inducible co-stimulator ligand (ICOS-L), programmed death-1 ligand (PD-L1), programmed death-2 ligand (PD-L2), B7-H3 and B7-H4 were determined by FACS analysis. The results demonstrate that PMT^wt^ alone activates monocytes only to a small extent as can be seen by the nearly unchanged expression patterns compared to the unstimulated samples. Samples that had been treated with LPS and PMT^wt^ showed an increase in the level of CD80 when compared to the LPS-treated control and a smaller increase was also detectable for B7H3, B7H4 and MHC2 (Figure 1A). Stimulation with the inactive PMT^RBD^ did not change the expression pattern of untreated and LPS-treated hBDMs at all (data not shown). We therefore conclude that PMT^wt^ by itself is unable to stimulate monocyte activation and that it only marginally increases monocyte activation induced by LPS.

Next, we investigated, whether PMT modulates the inflammatory response of monocytes by inducing or inhibiting their ability to release cytokines. We stimulated cells with PMT^wt^ or inactive PMT^RBD^ alone or in combination with LPS, as described before, and measured the release of pro-inflammatory IL-6, TNF-α and IL-12p40 and anti-inflammatory IL-10 by ELISA analyses (Figure 1B). As expected, LPS-stimulated monocytes released elevated levels of all four cytokines. PMT^wt^ only enhanced the secretion level of IL-6 in a statistically relevant manner. While the toxin had a minor influence on LPS-triggered TNF-α secretion and IL-10 production, it elevated the LPS-induced IL-6 level considerably, but almost totally abrogated the IL-12p40 release of activated cells. Since the truncated toxin again did not affect hBDMs we assigned the observed modulation to the catalytic function of the toxin and continued the studies with PMT^wt^.

### PMT attenuates T cell-activating ability of LPS-activated monocytes

T cell activation by antigen presenting cells requires the interaction between MHC complexes and T cell receptors as well as the binding of costimulatory B7 molecules to the CD28 receptor on T cells [21]. Additionally, IL-12 exerts a strong synergistic effect with the B7/CD28 interaction in inducing proliferation and cytokine production in T cells [22].

As PMT^wt^ only slightly elevated TLR4-mediated expression of costimulatory ligands on hBDMS (Figure 1A) and almost totally prevented LPS-stimulated IL-12p40 production (Figure 1B), we asked whether the toxin would affect T cell proliferation induced by the presence of LPS-treated hBDMs. To this end we performed mixed lymphocyte reactions with LPS- and/or PMT^wt^-stimulated monocytes and allogeneic T cells. The quantification of T cell proliferation after three days of co-culture revealed that PMT-stimulated monocytes did not differ significantly from unstimulated cells in mediating T cell proliferation. However, the toxin strongly inhibited the ability of LPS-stimulated hBDMs to activate T cell proliferation (Figure 1C). This pronounced suppression of T cell activation was reversed by adding recombinant IL-12 or supernatants from LPS-treated cells known to have a high level of IL-12p40 (Figure 1B and D2A). We therefore conclude that the suppressive influence of PMT^wt^ on monocyte-mediated T cell activation is due to a change in cytokine release, and in particular caused by the abrogated IL-12p40 production.

### PMT does not alter the cytokine release of LPS-activated hBDMs via the cyclooxygenase (COX)-2/Prostaglandin E2 (PGE2) pathway

**Figure 2:**
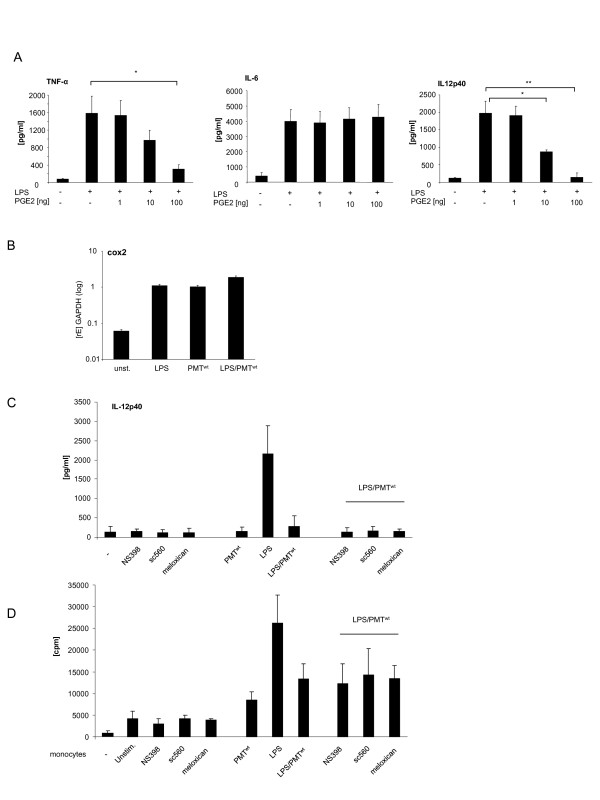
**Contribution of the COX-2/PGE2 pathway to the PMT-mediated modulation of LPS-activated monocytes.** A. Monocytes were stimulated overnight with LPS (30 ng/ml) and treated in parallel with PGE2 in increasing concentrations. Supernatants were used to quantify the release of TNF-α, IL-6 and IL-12p40 by ELISA. Presented is the mean and standard deviation of three independent experiments. B. Cells were stimulated with LPS (30 ng/ml; 1 h), PMT^wt^ (1 μg/ml; 4 h) or PMT^wt^/LPS (4 h/1 h) or left unstimulated. RNA was extracted, cDNA was prepared and used for quantitative PCR analysis with specific primers for COX2. The results are presented as relative expression to the reference gene (GAPDH). Shown is one representative experiment with 10% standard error. C. Monocytes were pre-treated for 2 hours with the cox-inhibitors NS398 (3.8 μM), sc560 (6.3 μM) or meloxican (4.7 μM). Then cells were stimulated with PMT^wt^ (1 μg/ml, 3 h prestimulation) and/or LPS (30 ng/ml; over night stimulation). The release of IL-12p40 was measured by ELISA. Shown is the mean of three independent experiments (mean ± SD; n = 3) D. Monocytes were stimulated as described in C. After washing, the cells were used for MLR with 1x10^4^ CD14^+^ hBDMs and 1x10^5^ CD3^+^ alloreactive T cells. After three days of co-culture, proliferation of T cells was analysed by [^3^H]-thymidine incorporation and presented as counts per minute (cpm). Statistical significance was calculated by using student’s *t* test with *P < 0.05 and **P < 0.01.

To identify the mechanism behind the PMT-modulated cytokine secretion of LPS-activated hBDMs, we first examined the COX/PGE2 pathway. The lipid mediator PGE2 is a well-established immunomodulatory substance which is produced during the immune response of antigen presenting cells. PGE2 is known to be a potent inducer of IL-10 and a strong inhibitor of IL-12 production [23,24]. COX proteins play a key role in the synthesis of prostaglandins. Whereas COX-1 is constitutively expressed and induces a basal level of PGE2, COX-2 is specifically induced by inflammatory stimuli [25]. To check whether PGE2 could be responsible for the PMT-induced blockade of IL-12p40, we treated LPS-stimulated hBDMs with increasing concentrations of recombinant PGE2 and quantified the release of cytokines. Whereas there was no significant influence detectable on IL-6 production, LPS-induced TNF-α and IL-12p40 secretion were diminished by PGE2 (Figure 2A). We next determined the mRNA level of COX-2 in LPS and/or PMT^wt^-treated hBDMs to investigate whether PGE2 could be released in this setting. The performed quantitative real-time PCRs showed that LPS and PMT^wt^ both triggered COX-2 gene transcription but that there was no pronounced synergistic effect between PMT and LPS (Figure 2B). To investigate whether the minor change in COX-2 expression could explain the PMT-induced change in cytokine release, we used various COX-2 inhibitors. Neither SC560 nor Meloxicam or NS398 neutralised the blockade of IL-12p40 production (Figure 2C) or changed the pattern of released IL-6 and TNF-α (data not shown). Mixed lymphocyte reactions additionally revealed that the COX-2 inhibitors did not restore the T cell-activating ability of LPS-treated monocytes after PMT^wt^-stimulation (Figure 2D). Based on these data we exclude a prominent impact of the COX2/PGE2 pathway on the PMT^wt^-mediated effect on endotoxin-stimulated hBDMs.

### PMT-induced modulation of LPS-triggered IL-12 release involves the Gαi/cAMP pathway

**Figure 3:**
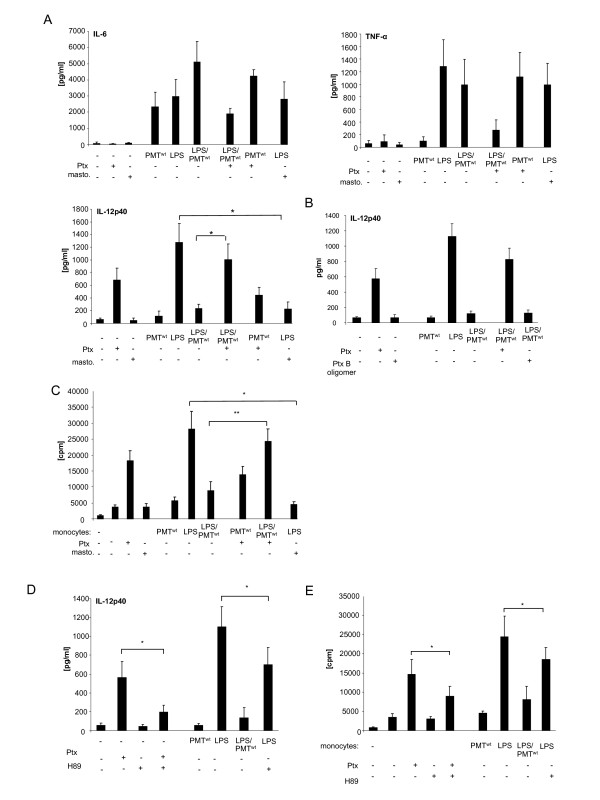
**Gα**_**i**_**-mediated modulation of LPS-induced IL-12 production.** A. Monocytes were stimulated overnight with Pertussis toxin (Ptx; 200 ng/ml) or left unstimulated. The next day PMT^wt^ (1 μg/ml) or mastoparan (20 μM) was added. After three hours LPS was added for overnight incubation (30 ng/ml). The release of TNF-α, IL-6 and IL-12p40 were measured by ELISA. Shown is the mean of three independent experiments (mean ± SD; n = 3). B.As additional control cells were treated with Ptx B oligomer and ELISAs were performed with collected supernatant. (mean ± SD; n = 2) C. Monocytes were stimulated as described in A. After washing, co-cultures of CD14^+^ hBDMs and CD3^+^ alloreactive T cells were prepared. After 3 days proliferation of T cells was analysed by [^3^H]-thymidine incorporation and presented as counts per minute (cpm), mean ± SD; n = 3. D. Cells were stimulated as followed: H89 (10 μM; 2 h pre-stimulation) and LPS (30 ng/ml) or Ptx (200 ng/ml) overnight, PMT^wt^ (1 μg/ml, 3 h pre-stimulation) and LPS overnight. Supernatants of cells were used for IL-12 ELISAs. The results are presented as mean ± SD; n = 3. E. Equally treated monocytes were used for MLRs with alloreactive T cells. Shown is the mean and standard deviation of three independent experiments. Statistical significance was assessed by using student’s *t* test with *P < 0.05 and **P < 0.01.

In order to characterize the PMT-induced signalling pathway that modulates cytokine production of LPS-activated monocytes, we next focused on the heterotrimeric G protein Gα_i_ that is directly activated by PMT [10]. While the exact mechanism is still under investigation, it is commonly accepted that Gα_i_- and Gα_s_-mediated alterations in intracellular cAMP metabolism play a key role in modulating IL-12 production. At this, GPCR ligands of adenylate cyclase-activator Gα_s_ as well as GPCR agonists of adenylate cyclase-inhibitor Gα_i_ were shown to decrease the capacity of antigen presenting cells to produce IL-12 [8]. Whereas the connection of adenylate cyclase-activation and IL-12 decrease may be more generally accepted, investigations with the Gα_i_-activator mastoparan, the Gα_i_-inhibitor Ptx as well as studies using Gα_i_-deficient mice clearly revealed an inhibitory effect of this G protein on LPS-induced cytokine release in different cells types, including human monocytes [26-28]. To investigate the role of Gα_i_ on the PMT-mediated manipulation of TLR4 signalling we pre-treated LPS and/or PMT^wt^-stimulated monocytes with Ptx or mastoparan. The performed cytokine ELISAs show that neither Ptx nor mastoparan significantly influenced IL-6 production of unstimulated or stimulated cells. The alteration of TNF-α was more influenced but statistically not significant. The impact of Gα_i_ was most pronounced on IL-12p40. Mastoparan mimicked the inhibition of IL-12p40 and Ptx could almost fully restore the LPS-induced production of IL-12p40 after toxin-treatment (Figure 3A). To confirm that the Ptx-mediated reconstitution of IL-12 production is due to the influence of G proteins, we included the B oligomer subunit of Ptx in our studies. The Ptx B oligomer is the receptor binding domain but can also initiate signal transduction pathways [29]. However, it does not play a role in the activation of G-protein signalling that is initiated by the catalytic subunit. As it exerted no influence on the PMT-mediated suppression of IL-12p40 (Figure 3B), we assigned the Ptx effect to the inhibition of Gα_i_.

Moreover, in mixed lymphocyte reactions using alloreactive T cells it was revealed that Ptx reconstituted the PMT^wt^-blocked activation of T cells proliferation through LPS-stimulated monocytes, while mastoparan/LPS-treated monocytes on the other hand were less activating than cells treated with LPS only (Figure 3B). These data implicate that the PMT^wt^-activated Gα_i_ protein is responsible for the abolishment of LPS-activated IL-12p40 release, while its impact on IL-6 and TNF-α is only marginal.

In order to further analyse the link between PMT^wt^-activated Gα_i_ and IL-12p40 suppression, we next concentrated on protein kinase A (PKA), as elevated cAMP concentrations can result in PKA activation [30]. This serine/threonine kinase mediates phosphorylation of substrates and subsequently induces the transcription of a variety of genes via the binding of transcription factors such as CRE-binding protein (CREB) to the DNA binding motif cAMP-response element (CRE) present in the promoter region of cAMP-regulated genes [31,32]. To mimic the PMT-induced inhibition of PKA caused by an inhibition of adenylate cyclase and cAMP accumulation, we included the PKA-specific inhibitor H89 in our studies. As a control, we verified that the inhibitor was able to block Ptx-induced IL-12p40 production (Figure 3C). Compared to PMT^wt^, H89 did not totally abolish LPS-induced IL-12p40 release but significantly diminished the LPS-induced release of the cytokine (Figure 3C) and as a consequence decreased the T cell-activating ability of hBDMs (Figure 3D). These data indicate a prominent role for PKA in the suppression of LPS-induced IL-12p40 production.

### PMT-activated JNK contributes to the suppression of IL-12 production

**Figure 4:**
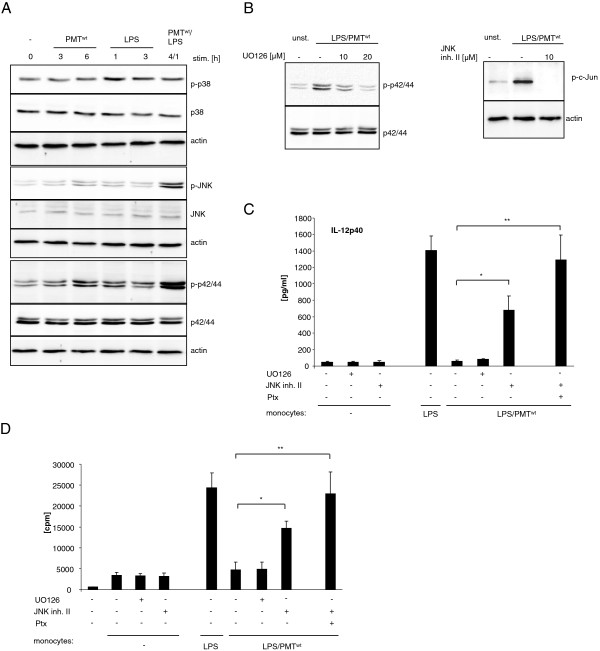
**Involvement of MAPkinases in the modulation of LPS-induced IL-12 release.** A. Monocytes were stimulated with PMT^wt^ (1 μg/ml) and/or LPS (30 ng/ml) for the indicated time points or left unstimulated. The cells were lysed and extracted with RIPA buffer as described under Experimental procedures. Total amounts of p42/44, JNK and p38 and the phosphorylation levels of the MAP kinases was determined by subsequent immunoblotting with specific antibodies (p42/44: Thr202/Tyr204 JNK: Thr183/Tyr185, p38: Thr180/Tyr182). Shown is one representative blot from three. B. Cells were pre-treated for two hours with UO 126 or JNK inhibitor II (concentrations indicated) and then stimulated with PMT^wt^ (1 μg/ml, 4 h) and LPS (30 ng/ml, 1 h). Unstimulated cells were used as a control. Lysates of the cells were immunoblotted with antibodies against p-p42/44, p42/44, p-c-Jun, c-Jun or actin. C. Cells were pre-treated with Ptx (200 ng/ml, overnight), UO 126 (10 μM, 2 h) or JNK inhibitor II (20 μM, 2 h), followed by stimulation with PMT^wt^ (1 μg/ml, 3 h pre-treatment) and LPS (30 ng/ml) overnight. The release of IL-12p40 was measured by ELISA (mean ± SD; n = 2). D. Cells that were treated like described in C and used for MLR (1x10^4^ CD14^+^ hBDMs, 1x10^5^ CD3^+^ alloreactive T cells). After three days of co-culture proliferation of T cells was analysed by [^3^H]-thymidine incorporation and presented as counts per minute (cpm). Results are given as mean ± SD; n = 2. Statistical significance was calculated with a student’s *t* test with *P < 0.05 and **P < 0.01.

Next we investigated whether other known regulators of IL-12p40 may contribute to the PMT-induced abolishment of LPS-mediated IL-12p40 release. Mitogen activated protein kinases (MAP kinases) are described to modulate IL-12 production in myeloid cells. Whereas ERK (p42/44) and JNK were shown to inhibit IL-12p40 release [33,34], p38 is known as an inducer of IL-12p40 [35]. We therefore determined the activation status of ERK, JNK and p38 after PMT^wt^ treatment and the modulation of LPS-activated MAP kinases. The performed western blot analyses revealed that PMT, as expected, neither significantly activated p38 nor modulated the slight LPS-induced p38 phosphorylation. However, ERK activation was enhanced after six hours of PMT^wt^ treatment and the LPS-mediated phosphorylation was severely enhanced by the toxin. In addition, the low JNK activation detectable after six hours of PMT^wt^ and one hour of LPS-treatment was strongly enhanced by simultaneous stimulation with both stimuli (Figure 4A). To verify whether this observed activation of ERK and JNK could account for the suppression of LPS-mediated IL-12p40 production, we used specific MAP kinase inhibitors. Monocytes were pre-treated with the MEK1 inhibitor UO126 (upstream of ERK) or JNK inhibitor II and stimulated with PMT^wt^ and LPS. The blots in Figure 4B show that 20 μM of UO 126 reduced LPS/PMT^wt^-induced ERK phosphorylation to basal levels and 10 μM of JNK inhibitor II totally blocked the activation of its downstream target c-Jun. ELISAs performed with supernatants of these cells demonstrated that while the inhibition of ERK did not affect LPS-induced IL-12p40 release, the suppression of JNK restored LPS-induced cytokine production (Figure 4C). Simultaneous inhibition of JNK and Gα_i_ activation through Ptx treatment abolished the PMT effect (Figure 4C) and restored the T cell-activating ability of LPS-treated cells after PMT^wt^-stimulation (Figure 4D). These data indicate that PMT inhibits LPS-mediated IL-12p40 expression via Gα_i_-mediated signalling and the activation of JNK.

### PMT-stimulated JNK-activation involves PI3-kinase

**Figure 5:**
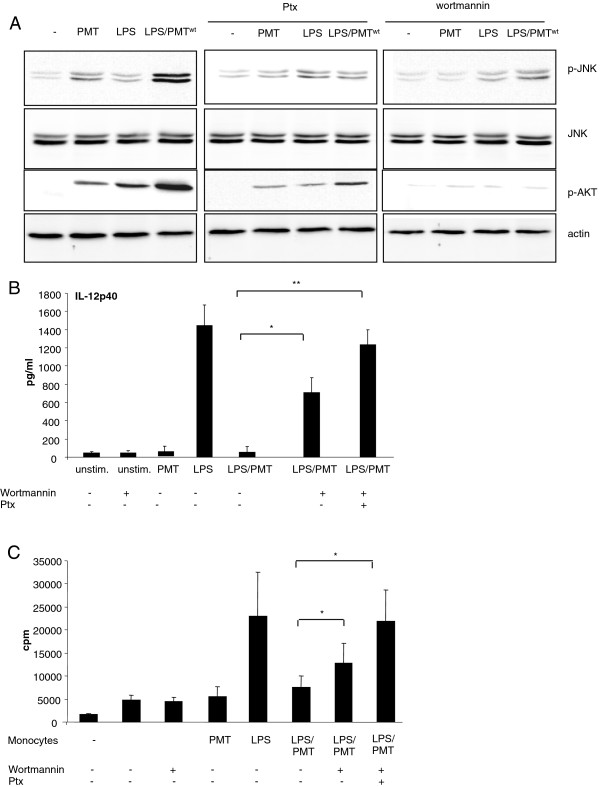
**Involvement of PI3kinase in JNK-activation.** A. Monocytes were pre-treated with Ptx (overnight incubation, 200 ng/ml) or wortmannin (2 h, 100 nM) and then stimulated with PMT^wt^ (1 μg/ml, 4 h) and or LPS (30 ng/ml, 1 h). Whole-cell lysates were prepared, proteins were separated by SDS-PAGE and p-JNK (Thr183/Tyr185), JNK, p-AKT (Thr308) and actin were detected using specific antibodies. Shown is one representative western blot out of three. B. Cells were treated as described in A or stimulated with Ptx and wortmannin. Supernatants were collected and IL-12p40 ELISAs were performed. Results are presented as mean ± SD; n = 3. C. Monocytes were stimulated like described in B and MLRs were performed. Results are given as mean ± SD; n = 3. Statistical significance in B. and C. was calculated with a student’s *t* test with *P < 0.05 and **P < 0.01.

Our next experiments addressed the question how PMT activates JNK. Recent publications suggest PI3 kinase as a mediator between Gα_i_-proteins and Ras/MAP kinase activation [36,37]. Interestingly, PMT activates PI3-kinase γ through dissociation of Gβγ subunits from the Gα_i_ subunit [38]. Here, we demonstrate by western blot analysis that PMT^wt^ induces the phosphorylation of the PI3-kinase target Akt in primary monocytes (Figure 5A). To check whether the activation of JNK depends on PI3-kinase activation we blocked PI3 kinase using wortmannin and observed a reduction of JNK phosphorylation in PMT^wt^- and most notably in LPS/PMT^wt^-stimulated cells (Figure 5A, right section). Furthermore, the inhibition of Gα_i_-activation by treatment with Ptx diminished the stimulated Akt activation downstream of PI3 kinase and impaired the activation of JNK (Figure 5A, middle section). This suggests that PI3 kinase mediates PMT^wt^-stimulated JNK activation and that the βγ subunits of Gα_i_ and other PMT-activated Gα proteins induce the activation of the kinase. ELISA experiments confirmed that the inhibition of JNK activation through wortmannin could reduce the PMT-mediated IL-12p40 suppression. Finally, the inhibition of both, JNK and Gα_i_-mediated signalling through Ptx, totally restored the LPS-induced production of the cytokine (Figure 5B). Performed MLRs confirmed the impact of the restored IL-12-production on the T cell-proliferation (Figure 5C).

**Figure 6:**
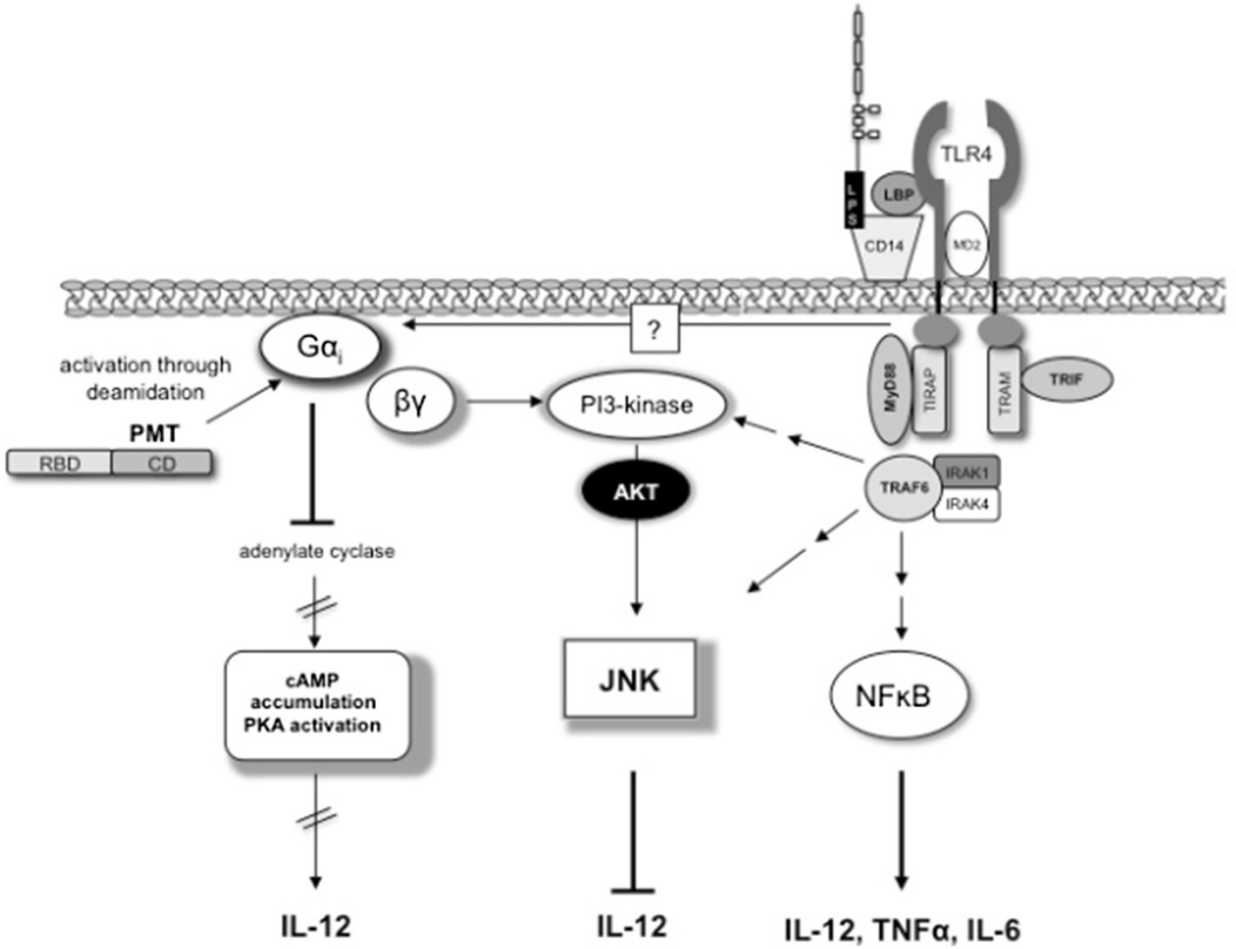
**Proposed model of PMT-induced inhibition of LPS-stimulated IL-12 release.** LPS stimulates the TLR4-mediated activation of the NFĸB-pathway and thereby the production of TNF-α, IL-6 and IL-12p40. PMT inhibits the production of IL-12 by Gα_i_-mediated inhibition of adenylate cyclase and cAMP accumulation and by Gβγ-mediated activation of PI3 kinase and JNK activation. PMT is shown with its N-terminal receptor binding domain (RBD) and C-terminal catalytic domain (CD).

Taken together we propose the following model: LPS stimulates TLR4-mediated activation of the NFĸB-pathway and modulates the production of TNF-α, IL-6 and IL-12p40. PMT inhibits the production of IL-12p40 through Gα_i_-mediated inhibition of adenylate cyclase and cAMP accumulation as well as by PI3kinase/Akt and JNK activation mediated by the dissociated Gβγ subunit (Figure 6).

## Discussion

Antigen presenting cells are crucial for the first-line host defence and act as mediators between innate and adaptive immunity. One major pathway of these cells involves TLR-mediated signalling events, which can detect and react to extracellular as well as intracellular microbial components [39,40]. To be effective, immune responses need the crosstalk between TLR-mediated and other signalling cascades that play a role in immune responses or other cellular pathways, respectively. These interactions can have synergistic effects but can also antagonise responses in order to prevent excessive inflammation. To modify and presumably escape the host’s immune response pathogens have developed various strategies to interfere with signalling cascades of the host [41,42]. Besides direct inhibition or modification of TLR signalling, induction of alternative regulative or antagonistic endogenous signalling cascades is another strategy in immune evasion. Here, we investigated how the activation of heterotrimeric G proteins through the bacterial toxin PMT modulates TLR4-mediated activation of human blood-derived monocytes and how this potentially affects cells of the adaptive immune system such as T cells.

Our data on the expression of MHC and costimulatory B7 family members show that PMT alone does not activate monocytes by itself and only slightly enhances LPS-triggered cell activation. However, PMT is able to modulate the TLR4-mediated cytokine production of these cells significantly leading to an almost complete block of IL-12p40 production. Bioactive IL-12 is a potent activator of natural killer (NK) cell and T cells and, together with the B7/CD28 interaction between antigen presenting cells and T cells, IL-12 has a strong synergistic effect in inducing T cell proliferation [22,43]. Our data show that due to the absence of IL-12, PMT suppresses T cell activation induced by LPS-activated hBDMs. This effect strictly depends on IL-12, as activity could be restored by adding IL-12-containing supernatants of LPS-stimulated hBDMs or recombinant IL-12 to the mixed lymphocyte reaction. Although IL-10 is a known suppressor of IL-12 activity [44], a dominant role of IL-10 could be excluded, since PMT only slightly enhanced the LPS-induced IL-10 production.

Bagley et al.. demonstrated that PMT also suppresses LPS-stimulated IL-12 release of human macrophage-derived dendritic cells and they speculate that this blockade is caused by Gα_q_-mediated Phospholipase C-β activation and subsequent intracellular calcium mobilization, but they did not further investigate this [45]. For human monocytes we suggest that PMT prevents TLR4-mediated IL-12p40 production through Gα_i_- and βγ-mediated signalling cascades, respectively. PMT activates Gα_i_ and thereby inhibits adenylate cyclase activity and cAMP accumulation, independently of Gα_q_ and Gα_12/13_[10]. Our data point to Gα_i_ signalling as the Gα_i_ agonist mastoparan mimicked the suppression of LPS-induced IL-12p40 production. Recently it became clear that data obtained from experiments using mastoparan have to be interpreted with care as mastoparan can bind the toxic lipid A portion of the LPS molecule [46]. Nevertheless, as in our studies mastoparan only blocked LPS-stimulated IL-12p40 production and did not block TLR4-mediated IL-6 and TNF-α expression, we assign the result primarily to Gα_i_-mediated signalling. Additionally, the Gα_i_-inhibitor Pertussis toxin (Ptx) reversed the PMT-mediated blockage of IL-12-release and restored the T cell-activating ability of LPS-treated monocytes.

In the past it has been shown that both Gα_i_ and Gα_s_, although having opposite function and acting as negative regulators for each other, are able to downregulate IL-12 production [8,47]. This suggests that alterations in intracellular cAMP metabolism in general play a role in modulating the IL-12 production. The molecular mechanism, however, is only poorly understood. Our data indicate that Protein kinase A (PKA) is involved in the PMT-mediated suppression of IL-12p40 production induced by LPS. In our experiments the PKA-specific inhibitor H89 significantly diminished LPS-induced release of IL-12p40 and decreased the T cell-activating ability of hBDMs. It thereby mimicked the PMT-induced effect on monocytes at least to some extent. H89 totally blocked Ptx-induced IL-12p40 production, demonstrating that the Gα_i_-protein-inhibitor Ptx induces IL-12 via PKA.

Our experiments suggest that signalling mechanisms directly dependent on Gα_i_ account partially for the PMT-triggered abrogation of LPS-induced IL-12p40 production, but they also indicate that other pathways are involved. Another signalling cascade connected to IL-12 production is the MAP kinase pathway, whereas ERK (p42/44) and JNK are known inhibitors of IL-12 production [35]. Our investigations demonstrate that simultaneous stimulation of the cells with PMT and LPS induced a strong activation of JNK. Furthermore, inhibition of JNK restored LPS-induced IL-12 production after PMT-treatment. Although G proteins are known to activate the MAP kinase cascade, the exact mechanism is not well defined. However, a potential link to known PMT-mediated signalling pathways comes from a publication that showed the activation of this pathway was induced by phospholipase C and protein kinase C [48]. These proteins are known to be activated by PMT through Gα_q_ activation [16]. Additionally, βγ subunits dissociated from their Gα_i_ subunit were also identified as initiators of the Ras/MAP kinase pathway [37,49]. Our experiments point to Gα_i_ proteins as well as their respective dissociated βγ subunits as mediators of the PMT-stimulated JNK phosphorylation, as the Gα_i_ inhibitor Ptx significantly diminished the PMT/LPS-induced activation of the kinase. It is also possible that βγ subunits of other PMT-activated G proteins may additionally be involved, as the inhibition of Gβγ release through Ptx did not completely suppress JNK activation. The possibility that βγ subunits of various G proteins are involved has also been discussed previously by Preuss et al.. [38].

Kranenburg et al. claim from their experiments in COS cells and fibroblasts that the missing link between Gβγ and Ras/MAP kinase activation is PI3 kinase [37]. In addition, Lopez-Ilasaca et al. confirmed that overexpressed PI3 kinase activates MAP kinases [36,37]. Recent studies showed that PMT activates PI3 kinase γ through Gβγ subunits [38,50] and our current investigation confirms the finding that the toxin mediates the phosphorylation of Akt, the target of PI3 kinase, also in primary monocytes. The publication of La Sala et al. suggests that the Gα_i_-protein activator C5a suppresses TLR4-mediated IL-12 via the Akt pathway and JNK, but independently of PI3 kinase [34]. We show here that in the case of PMT-mediated Gβγ activation the PI3 kinase inhibitor wortmannin diminished the activation of JNK, suggesting that there is a possible link between MAP kinase activation and PI3 kinase/Akt.

Our data clearly show that inputs from Gα_i_ protein-mediated signalling pathways modulate TLR4-induced signalling. Other authors even suggest a direct interaction between TLR4 and Gα_i_. Solomon et al. demonstrated that the LPS/LBP receptor CD14 co-immunoprecipitates with various α subunits of the Gα_i_/Gα_o_ family and that the Gα_i_ activator mastoparan inhibits LPS-induced IL-6 and TNF-α production in human monocytes [28]. Another report claims that Gα_i_ itself is activated rapidly but transiently by LPS through an unknown mechanism. In a mouse model the authors showed that Ptx increased the level of LPS-induced plasma inflammatory mediators, whereas mastoparan decreased the cytokine level [26]. However, in these studies the influence of Gα_i_ activation on IL-12p40 production as a downstream result of these signalling events had not been investigated. Recently, another report confirmed the hypothesis that TLR ligands activate Gα_i_ proteins in endothelial cells [51]. It was shown that LPS stimulates PI3 kinase and MAP kinase activation via Gα_i_, independently of the TLR-induced MyD88-TRAF6 pathway. The authors speculate that TLR2, 3 and 4 can also directly interact with Gα_i_ via their intracellular domains due to a consensus motif for Gα_i/0_ binding (Figure 6). In our hands LPS-stimulated Akt-activation could be diminished by Ptx which supports the hypothesis of LPS-induced Gα_i_ activation. Regardless of whether PMT is the only Gα_i_ activator or whether the toxin additionally increases the LPS-triggered Gα_i_ activation, our data clearly show that the PMT-induced Gα_i_-mediated signalling interferes with and suppresses TLR4-induced IL-12p40 production.

## Conclusion

In summary we show that PMT inhibits the TLR4-mediated production of IL-12p40 at least in two ways. First of all, PMT induces Gα_i_-mediated inhibition of adenylate cyclase and cAMP accumulation. Furthermore, PMT leads to a Gβγ-mediated activation of PI3kinase and subsequent JNK activation mediated by Gα_i_ and presumably other Gα subunits activated by PMT. These signalling cascades act specifically on the production of IL-12p40 as they influence the LPS-stimulated TNF-α release only slightly and have no impact on IL-6 release. Taken together our observations contribute to the understanding of the interaction between G protein-mediated and TLR4-mediated signalling and show how a bacterial toxin is able to manipulate monocytes which eventually results in impaired T cell activation.

## Methods

Recombinant PMT^wt^ and PMT^RBD^ were kindly provided by Dr. Joachim Orth and Prof. Dr. Dr. Klaus Aktories from Freiburg. The TLR agonist LPS was provided by U. Seydel (Borstel). The p44/42 inhibitor UO126, JNK inhibitor II, Pertussis toxin (full length and B oligomer subunit), mastoparan and wortmannin were purchased from Calbiochem. Recombinant IL-12 was obtained from Immunotools.

### Preparation of monocytes

Monocytes were isolated from fresh blood or buffy coat by density gradient centrifugation (Biocoll separating solution 1.077 g/mL; Biochrom AG) and washed three times with PBS. CD14 cells were positively selected by magnetic-associated cell sorting (AutoMACS: program possel; Miltenyi Biotec). Sorted cells were cultured in RPMI 1640 medium (Biochrom AG) supplemented with 10% FBS (BioWest) and 1% penicillin and streptomycin (PAA) at 37°C in a humidified atmosphere in the presence of 5% CO_2_.

### Mixed lymphocyte reaction (MLR)

T cells were obtained from fresh blood by positive selection with CD3-labelled magnetic beads and FcR blocking reagent (AutoMACS: program possel; Miltenyi Biotec).

MLRs were performed in 96 well plates in allogeneic settings: purified T cells (1x10^5^) were co-cultured with pre-treated and washed monocytes (1x10^4^). Cells were cultured for 3 days and exposed to [^3^ H]-thymidine (Amersham Pharmacia Biotech GmbH) during the last 6 h of culture. Thymidine uptake was measured using a liquid scintillation counter.

### Flow cytometry (FACS)

Briefly, cells were washed with PBS/10% FBS, blocked for 30 min in PBS/10% FBS, and incubated with the corresponding antibodies (anti-B7H3-PE, anti-B7H4-PE, anti-PDL-1-APC, anti-PDL-2PE, anti-ICOSL-PE from eBioscience anti-CD80-PE, anti-CD86-PE, anti-MHC2-FITC from BD Pharmingen) in PBS/10% FBS for 1 h at 4°C. After three washes quantification was done by FACS analysis using a BD FACSCanto workstation and BD FACSDiva software (BD Biosciences). Overlays were produced using Weasel v2.5 software.

### ELISA

Cell-free supernatants were harvested and analysed for IL-6, IL-12p40, IL-10 and TNF-α by commercial available ELISA kits (OptEIA; BD).

### Western blotting

After washing with PBS, cells were resuspended in RIPA buffer (150 mM NaCl, 1 mM EDTA, 1% Triton X-100, 1% Deoxycholat, 1 mM sodium fluoride, 25 mM Tris) containing phosphatase inhibitors (PhosSTOP, Roche Diagnostics) and incubated for 45 min at 4°C. After centrifugation, lysates were used as whole-cell extracts. Samples were separated on 10% SDS-PAGE, transferred to nitrocellulose and incubated with the relevant antibodies (Cell Signaling Technology: p42/44, pThr202/Tyr204 p42/44, JNK, pThr183/Tyr185 JNK, p38, pThr180/Tyr182 p38, pSer63 c-Jun, pThr 308 Akt, Actin) followed by incubation with the respective HRP-coupled antibody (Cell Signaling Technology) and detection by enhanced chemiluminescence.

### Reverse transcription-PCR

RNA was extracted with the “High pure RNA Isolation Kit” (Roche). cDNA was prepared using ‘Reverse Aid First Strand cDNA Synthesis Kit’ (Fermentas life science). Aliquots of the cDNA were used for quantitative PCR analysis with the ‘SYBR Green Rox mix’ (Thermo Scientific) with the following primers: GAPDH, sense, 5′-acg gat ttg gct-3′,antisense, 5′-ttg acg gtg cca tgg aat ttg-3′, Cox2, sense, 5′-caa ctc tat att gct gga aca tgg a −3′,antisense, 5′-tgg aag cct gtg ata ctt tct gta ct-3′. The results were analysed using the Real-Time PCR System (Applied Biosystems). All results were normalized to the reference gene GAPDH.

### Statistics

Statistical significance was assessed by using student’s *t* test with *P < 0.05 and **P < 0.01.

## Competing interests

The authors declare that they have no competing interests.

## Authors’ contributions

DH carried out the experiments and wrote the manuscript. AS was involved in the generation of several data. SJW helped with the MLRs. KH conceived of the study, participated in its development and coordination and revised the manuscript. KFK participated in the design of the study and wrote the manuscript. All authors read and approved the final manuscript.
